# Supplementary material for: The Matrix Protein Cysrichin, a Galaxin-like Protein from Hyriopsis cumingii, Induces Vaterite Formation In Vitro
Source: Biology (Basel). 2023 Mar 15;12(3):447. doi: 10.3390/biology12030447 (PMC10045328; doi:10.3390/biology12030447)
Supplement: Supplementary file 1 [file biology-12-00447-s001.zip › File S1.pdf]

	A	B	C	D	E	F	G	I	J
1		Well	Fluor	Target	Content	Sample	Cq	t	tt
2		A07	SYBR	gill	Unkn	Experiment	34.20	10.3777060810274	-6.4878939189726
3		A08	SYBR	gill	Unkn	Experiment	34.09	10.3631708495188	-6.5024291504812
4		A09	SYBR	gill	Unkn	Experiment	33.82	10.2627439550829	-6.6028560449171
5		A10	SYBR	gill	Unkn	internal reference	23.83		
6		A11	SYBR	gill	Unkn	internal reference	23.73		
7		A12	SYBR	gill	Unkn	internal reference	23.56		
8		B07	SYBR	adductor muscle	Unkn	Experiment	30.85	12.8267379014297	-4.0388620985703
9		B08	SYBR	adductor muscle	Unkn	Experiment	30.72	12.9705813825016	-3.8950186174984
10		B09	SYBR	adductor muscle	Unkn	Experiment	30.46	12.9379203618099	-3.9276796381901
11		B10	SYBR	adductor muscle	Unkn	internal reference	18.02		
12		B11	SYBR	adductor muscle	Unkn	internal reference	17.75		
13		B12	SYBR	adductor muscle	Unkn	internal reference	17.53		
14		C07	SYBR	gonad	Unkn	Experiment	35.15	16.0594606641686	-0.8061393358314
15		C08	SYBR	gonad	Unkn	Experiment	34.99	16.3090204124296	-0.5565795875704
16		C09	SYBR	gonad	Unkn	Experiment	34.90	16.2707445285632	-0.5948554714368
17		C10	SYBR	gonad	Unkn	internal reference	19.09		
18		C11	SYBR	gonad	Unkn	internal reference	18.68		
19		C12	SYBR	gonad	Unkn	internal reference	18.63		
20		D07	SYBR	Hepatopancreas	Unkn	Experiment	33.57	16.2061949899017	-0.6594050100983
21		D08	SYBR	Hepatopancreas	Unkn	Experiment	33.42	16.0664658183303	-0.7991341816697
22		D09	SYBR	Hepatopancreas	Unkn	Experiment	32.76	15.4514216625072	-1.4141783374928
23		D10	SYBR	Hepatopancreas	Unkn	internal reference	17.36		
24		D11	SYBR	Hepatopancreas	Unkn	internal reference	17.35		
25		D12	SYBR	Hepatopancreas	Unkn	internal reference	17.31		
26		E07	SYBR	Food	Unkn	Experiment	29.96	12.8177935585978	-4.0478064414022
27		E08	SYBR	Food	Unkn	Experiment	29.62	12.5168694119019	-4.3487305880981
28		E09	SYBR	Food	Unkn	Experiment	29.53	12.4638759714755	-4.4017240285245
29		E10	SYBR	Food	Unkn	internal reference	17.14		
30		E11	SYBR	Food	Unkn	internal reference	17.11		
31		E12	SYBR	Food	Unkn	internal reference	17.06		
32		F07	SYBR	Mantle	Unkn	Experiment	25.57	8.7058669480360	-8.1597330519640
33		F08	SYBR	Mantle	Unkn	Experiment	25.43	8.6533645798985	-8.2122354201015
34		F09	SYBR	Mantle	Unkn	Experiment	25.37	8.6667416858385	-8.1988583141615
35		F10	SYBR	Mantle	Unkn	internal reference	16.87		
36		F11	SYBR	Mantle	Unkn	internal reference	16.77		
37		F12	SYBR	Mantle	Unkn	internal reference	16.70		
38									
39									
40									
41									
42									
43									
44									

	K	L	M	N	O	P	Q	R	S	T	U
1	ttt										
2	89.75335226	92.53787804	4.061363408								
3	90.66219281										
4	97.19808904										
5											
6											
7											
8	16.43685182	15.51054524	0.820086652								
9	14.87707106										
10	15.21771283										
11											
12											
13											
14	1.748526106	1.576541823	0.15024933								
15	1.470778087										
16	1.510321275										
17											
18											
19											
20	1.579431107	1.994855572	0.585960567								
21	1.740056536										
22	2.665079071										
23											
24											
25											
26	16.53907267	19.3504926	2.46441691								
27	20.37503437										
28	21.13737076										
29											
30											
31							gill	GI		89.7534	90.6622
32	285.9725877	292.1260321	5.501973298				adductor m	AM		16.4369	14.8771
33	296.5713492						gonad	G		1.7485	1.4708
34	293.8341593						Hepatopanc	H	Hepatopanc	1.5794	1.7401
35							Food	F		16.5391	20.375
36							Mantle	M		285.9726	296.5713
37											
38											
39							adductor m	15.5105	0.8201		
40							gill	92.5379	4.0614		
41							Mantle	292.126	5.502		
42							gonad	1.5765	0.1502		
43							Food	19.3505	2.4644		
44							Hepatopanc	1.9949	0.586		

	V	W	X	Y
1				
2				
3				
4				
5				
6				
7				
8				
9				
10				
11				
12				
13				
14				
15				
16				
17				
18				
19				
20				
21				
22				
23				
24				
25				
26				
27				
28				
29				
30			平均数	
31	97.1981		92.5379	4.0614
32	15.2177		15.5105	0.8201
33	1.5103		1.5765	0.1502
34	2.6651		1.9949	0.586
35	21.1374		19.3505	2.4644
36	293.8342		292.126	5.502
37				
38				
39				
40				
41				
42				
43				
44				
