# Supplementary figures and images for: The Matrix Protein Cysrichin, a Galaxin-like Protein from Hyriopsis cumingii, Induces Vaterite Formation In Vitro
Source: Biology (Basel). 2023 Mar 15;12(3):447. doi: 10.3390/biology12030447 (PMC10045328; doi:10.3390/biology12030447)

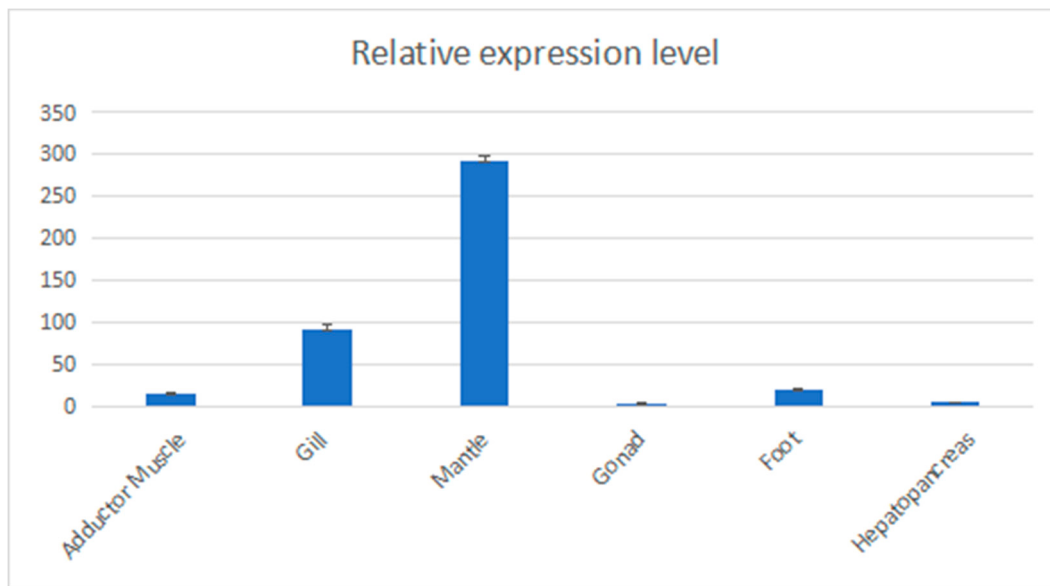

Figure S1. Tissue expression analysis of cysrichin.

Supplement: Supplementary file 1 [file biology-12-00447-s001.zip › Figure S1.pdf]
